# 235. Outcomes Associated with Extended Oral Antibiotic Prophylaxis After 2-Stage Exchange Surgery to Prevent Recurrent Prosthetic Joint Infection

**DOI:** 10.1093/ofid/ofab466.437

**Published:** 2021-12-04

**Authors:** Marin L Schweizer, Poorani Sekar, Brice Beck, Bruce Alexander, Kelly Richardson, Daniel Suh, Hiroyuki Suzuki, Aaron J Tande, Mireia Puig-Asensio, Kimberly Dukes, Julia Walhof, Andrew Pugely, Christopher Richards, Stacey Hockett Sherlock, Rajeshwari Nair

**Affiliations:** 1 University of Iowa Carver College of Medicine, Iowa City, Iowa; 2 University of Iowa, Iowa City, Iowa; 3 Iowa City VA Health Care System, Iowa City, Iowa; 4 University of Iowa Hospitals and Clinics, Iowa City, Iowa; 5 Mayo Clinic, Rochester, MN; 6 University of Iowa Hospitals & Clinics, Iowa City, IA; 7 Iowa City VA, Iowa City, Iowa; 8 University of Iowa Hospital and Clinics, Iowa City, Iowa; 9 VA Iowa City Health Care System and University of Iowa, Iowa City, Iowa; 10 The University of Iowa Carver College of Medicine, Iowa City, Iowa

## Abstract

**Background:**

2-stage exchange (2SE) surgery is often used to treat chronic prosthetic joint infections (PJI). IDSA guidelines do not recommend oral antibiotic suppression after 2SE. However, a recent randomized trial suggested that oral antibiotics for 3 months after arthroplasty reimplantation may prevent recurrent PJI. Objective: To compare rates of treatment failure (i.e., recurrent PJI) and adverse reactions (ARs) among patients who received < 1 month of antibiotics directly after reimplantation to those who received 1-3 months of antibiotics following reimplantation (extended antibiotics).

**Methods:**

This retrospective cohort study included patients with hip, knee, or shoulder PJI who underwent 2SE at 83 VA hospitals between the years 2003-2017. PJI was defined using administrative codes and microbiology data. Patients were followed for 5 years to assess treatment failure (TF) and ARs. TF was defined as recurrent PJI, debridement, or reoperation. ARs included *Clostridioides difficile* infections (CDI), or antibiotic associated diarrhea (AAD) during or 72 hours after antibiotics. Chi-square tests were used to compare outcomes. Cumulative incidence function curves were created to compare TF rates between those who did and did not receive extended antibiotic treatment, incorporating the competing risks of TF and death.

**Results:**

Of the 433 patients, most (97%) received < 1 month of oral antibiotics and 3% received extended antibiotics. The 15 patients who received extended antibiotics had similar rates of TF and ARs compared with patients who received < 1 month of oral antibiotics (Table). However, there was a trend toward higher rates of CDI (6.7% vs. 3.8%) and AAD (13.3% vs. 9.6%) among those who received extended antibiotics. There was no difference in TF comparing extended antibiotics with < 1 month of antibiotics, accounting for death (Figure).

Table: Treatment Failure and Adverse Reactions Among Those Who Did and Did Not Receive Extended Antibiotics

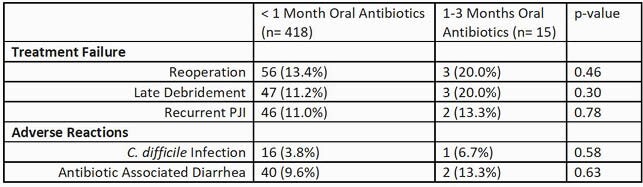

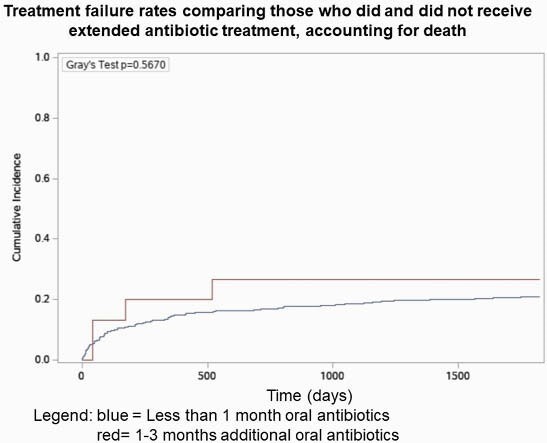

**Conclusion:**

Few patients received extended oral antibiotics in the study period. There were no statistically significant differences in TF or ARs between the 2 groups. Yet, there was a trend toward higher rates of ARs among the extended antibiotic group. Future prospective studies should assess both the potential benefits and ARs associated with extended antibiotics among patients undergoing 2SE surgery.

**Disclosures:**

**Marin L. Schweizer, PhD**, **3M** (Grant/Research Support)**PDI** (Grant/Research Support) **Bruce Alexander, PharmD**, **Bruce Alexander Consulting** (Independent Contractor) **Daniel Suh, MS MPH**, **General Electric** (Shareholder)**Merck** (Shareholder)**Moderna** (Shareholder)**Smile Direct Club** (Shareholder) **Aaron J. Tande, MD**, **UpToDate.com** (Other Financial or Material Support, Honoraria for medical writing) **Andrew Pugely, MD, MBA**, **Globus Medical** (Research Grant or Support)**Medtronic** (Consultant)**United Healthcare** (Consultant)

